# Early immune responses have long-term associations with clinical, virologic, and immunologic outcomes in patients with COVID-19

**DOI:** 10.21203/rs.3.rs-847082/v1

**Published:** 2022-02-02

**Authors:** Zicheng Hu, Kattria van der Ploeg, Saborni Chakraborty, Prabhu Arunachalam, Diego Mori, Karen Jacobson, Hector Bonilla, Julie Parsonnet, Jason Andrews, Haley Hedlin, Lauren de la Parte, Kathleen Dantzler, Maureen Ty, Gene Tan, Catherine Blish, Saki Takahashi, Isabel Rodriguez-Barraquer, Bryan Greenhouse, Atul Butte, Upinder Singh, Bali Pulendran, Taia Wang, Prasanna Jagannathan

**Affiliations:** University of California, San Francisco; Stanford University; Stanford University; Stanford University; Stanford University; Stanford University; Stanford University; Stanford University, Stanford; Stanford Medicine; Stanford University; Stanford University; Stanford University; Stanford University; JCVI; Stanford University; University of California, San Francisco; UCSF; UCSF; Bakar Institute for Computational Health Sciences, University of California, San Francisco; Stanford University School of Medicine; Stanford University School of Medicine; Stanford University; Stanford University

## Abstract

The great majority of SARS-CoV-2 infections are mild and uncomplicated, but some individuals with initially mild COVID-19 progressively develop more severe symptoms. Furthermore, there is substantial heterogeneity in SARS-CoV-2-specific memory immune responses following infection. There remains a critical need to identify host immune biomarkers predictive of clinical and immunologic outcomes in SARS-CoV-2-infected patients. Leveraging longitudinal samples and data from a clinical trial in SARS-CoV-2 infected outpatients, we used host proteomics and transcriptomics to characterize the trajectory of the immune response in COVID-19 patients within the first 2 weeks of symptom onset. We identify early immune signatures, including plasma RIG-I levels, early interferon signaling, and related cytokines (CXCL10, MCP1, MCP-2 and MCP-3) associated with subsequent disease progression, control of viral shedding, and the SARS-CoV-2 specific T cell and antibody response measured up to 7 months after enrollment. We found that several biomarkers for immunological outcomes are shared between individuals receiving BNT162b2 (Pfizer–BioNTech) vaccine and COVID-19 patients. Finally, we demonstrate that machine learning models using 7–10 plasma protein markers measured early within the course of infection are able to accurately predict disease progression, T cell memory, and the antibody response post-infection in a second, independent dataset.

## Introduction

The great majority of severe acute respiratory syndrome-related coronavirus 2 (SARS-CoV-2) infections initially present with mild to moderate symptoms. However, some patients with initially mild infections progress to more severe disease requiring hospitalization, and/or prolonged symptoms leading to sustained disability^1^. Early identification of these patients would help guide treatment decisions, including the use of monoclonal antibodies and novel antivirals that can prevent disease progression. Moreover, mild infections are an important contributor to ongoing viral transmission, and there is substantial heterogeneity in the degree of the SARS-CoV-2-specific memory immune response following infection^2–4^. An improved understanding of host determinants of clinical, virologic, and immunologic outcomes of SARS-CoV-2 infection can help spur the development of novel therapeutic and vaccination strategies.

The early host response to acute SARS-CoV-2 infection likely plays a critical role in determining disease outcome and generation of virus-specific memory immune responses. Nucleic acid pattern recognition receptors (PRRs) mediate the early detection and host response to viral infections, with RNA virus recognition thought to occur mainly in the endosomal and/or cytosolic compartment by two different PRRs: Toll-like receptors (TLRs) and RIG-I-Like Receptors (RLRs). Viral recognition by TLRs and RLRs typically triggers a signaling cascade leading to induction of pro-inflammatory cytokines and type I and type III interferons (IFN), which provide both a cell-intrinsic state of viral resistance and help coordinate the generation of adaptive immune responses^5^. Most studies evaluating the early host response have been cross-sectional, and/or performed in patients already with severe disease^6–13^; longitudinal studies among those presenting earlier in disease, with prospective clinical outcomes, are lacking^14^.

Following initial infection, SARS-CoV-2-specific memory immune responses results in protection from reinfection, likely mediated in part by SARS-CoV-2-specific memory T cell and both binding and neutralizing antibody responses^15–18^. However, there is considerable heterogeneity in the T cell and antibody response following natural SARS-CoV-2 infection, with rapid decay in early convalesce, providing valuable opportunities to identify key immune components that are associated with the establishment and durability of memory immune responses. Although mRNA vaccination also leads to establishment of protective immunologic memory^19^, this memory wanes^20^. Comparing responses induced by vaccination with those induced by natural SARS-CoV-2 infection could potentially guide researchers to better understand determinants of durable protective immunity and improve vaccine design.

In this paper, we utilized a multi-omics approach to define early infection signatures following SARS-CoV-2 infection that predict subsequent disease progression, oropharyngeal viral load, and SARS-CoV-2-specific memory immune responses. We leveraged longitudinal samples collected from outpatients enrolled in a randomized controlled trial of a type III interferon, Peginterferon Lambda-1a (Lambda, NCT04331899)^21^. In this trial, outpatients with initially mild to moderate COVID-19 were recruited within 72 hours of diagnosis, and followed through 7 months post-infection. We observed sequential activation of immune modules in initially mild to moderate COVID-19 patients within the first 2 weeks of symptom onset, including interferon responses, T cell activation and B cell responses. We identified variations in plasma proteins, early interferon signaling, and downstream cytokines (MCP1, MCP-2 and MCP-3) that were associated with multiple patient outcomes, including disease progression, viral load, memory T cell activity and S protein-binding IgG levels measured up to 7 months after enrollment. We also compared the immune response in COVID-19 patients to the response following COVID-19 mRNA vaccination, and identify biomarkers for immunological outcomes, including CXCL10, MCP-1 and Interferon gamma, that are shared between individuals receiving BNT162b2 (Pfizer–BioNTech) vaccine and COVID-19 patients. Finally, we demonstrate that a machine learning model using 7–10 plasma protein markers is able to accurately predict disease progression and the magnitude of the SARS-CoV-2 specific CD4+ T cell response and antibody response in a second, independent cohort.

## Results

### Transcriptomic and proteomic profiles correlate with the time to symptom onset in COVID-19 patients.

We recruited 108 participants with initially mild to moderate COVID-19 at diagnosis into this study. The median age of participants was 37 years (range 18–71) with 57% male, and 62% of Latinx ethnicity (1). Eight (6.7%) participants were asymptomatic at baseline. Of those with symptoms, the median duration of symptoms prior to randomization was 5 days (IQR 3–6 days, Supplementary Table 1).

**Figure 1:**
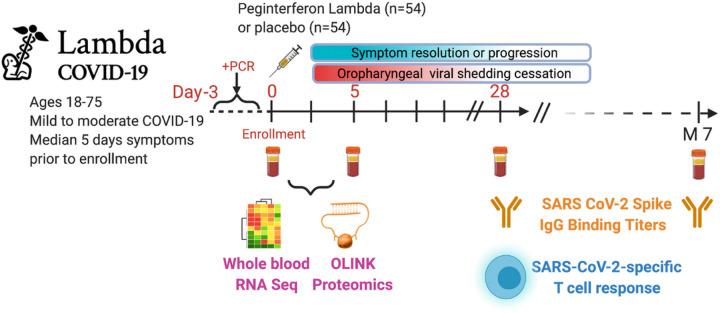
Study schema. Outpatients (n=108) with PCR-confirmed SARS-CoV-2 infection and swab obtained within 72 hours of randomization were enrolled in a Phase 2 clinical trial of subcutaneous Peginterferon lambda vs. placebo. In-person follow-up visits were conducted at day 1, 3, 5, 7, 10, 14, 21, 28, and month 7 post-enrollment, with assessment of symptoms and vitals, and collection of oropharyngeal swabs for SARS-CoV-2 testing. Blood obtained at Day 0 and 5 were evaluated by whole blood transcriptomics (RNA Sequencing), plasma proteomics (Olink), and SARS-CoV-2 specific antibodies. Clinical outcomes assessed included duration of symptoms and duration of virologic shedding. Immunologic outcomes assessed including SARS-CoV-2-specific T cell responses at day 28, and antibody responses at day 28 and month 7. Created with biorender.com.

Subjects were randomized to receive a single dose of Peginterferon Lambda or placebo at their first visit and followed up to 7 months post-enrollment (Figure 1). The median duration of viral shedding post-enrollment was 7 days, and symptoms was 8 days, and this did not differ between participants randomized to Lambda compared with placebo^21^. To profile the immune response in these patients, we conducted whole blood RNA-sequencing and plasma protein profiling with multiplex Olink panels (inflammation and immune response panels, n=184 proteins) using blood samples collected at day 0 and day 5 after enrollment. We assessed SARS-CoV-2-specific CD4+ T cell responses by intracellular cytokine staining using PBMC collected at day 28 after enrollment. We also measured IgG binding titers against the SARS-CoV-2 full length spike protein (S) using plasma collected at day 0, day 5, day 28, and month 7 (Figure 1).

**Figure 2:**
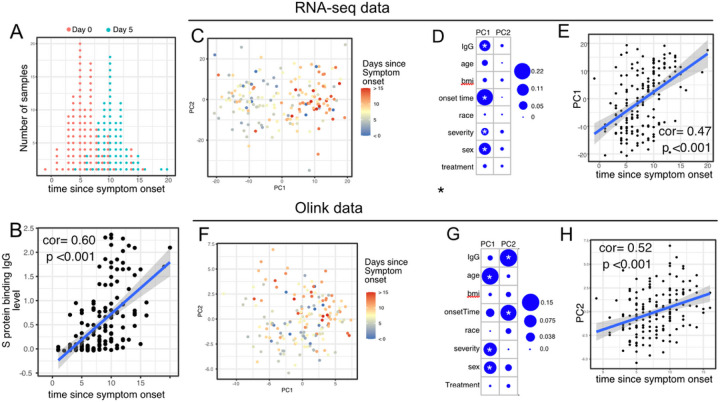
Transcriptomics and proteomics profiles correlate with the time to symptom onset in COVID-19 patients. (A) The distribution of RNA-seq sample collection time in respect to symptom onset. The colors of the dots represent the sample collection time from the enrollment. Asymptomatic cases are not shown. (B) Scatter plot showing the positive correlation between SARS-CoV-2 spike (S) protein binding IgG antibody level and the time since symptom onset. Pearson correlation is reported. (C) PCA plot of the RNAseq samples. The colors of the dots represent the sample collection time from the enrollment. (D) the percent of the variances of PC1 and PC2 explained by different clinical variables. Stars indicate FDR<0.05. (E) scatter plot showing the positive correlation between PC1 of the RNA-seq data and the time since symptom onset. Pearson correlation is reported. (F) PCA plot of the Olink proteomics data. The colors of the dots represent the sample collection time from the enrollment. (G) the percent of the variances of PC1 and PC2 explained by different clinical variables. Stars indicate FDR<0.05. (H) scatter plot showing the positive correlation between PC2 of the Olink data and the time since symptom onset. Pearson correlation is reported.

We first examined antibody levels and transcriptomic profiles at day 0 and day 5 after enrollment in both patients randomized to Peginterferon Lambda and placebo. Based on the subject-reported symptom starting date, samples at day 0 were collected a median 5 days after symptom onset (Range −1 to 15, Figure 2A). As expected, we observed a positive correlation between the S protein binding IgG levels at enrollment and the time since symptom onset (Figure 2B). We performed principal component analysis of transcriptomic data and calculated the correlation between the first two principal components (PC) and other clinical variables. We found that PC1 had the strongest association with the time since symptom onset and the IgG titer, suggesting that whole blood transcriptomic profiles capture the progression of the immune response in COVID-19 patients (Figure 2C–E). We also performed PCA analysis on the Olink data. Similar to results from the analysis of transcriptomics data, Olink data were associated with disease progression, as indicated by the high correlation between PC2 and the time since symptom onset (Figure 2F–H). We also observed an association between PC1 and age, which captures the impact of age on the plasma protein landscape in COVID-19 patients.

We previously reported that Peginterferon Lambda treatment neither shortened the duration of SARS-CoV-2 viral shedding nor improved symptoms in outpatients with COVID-19^21^. PCA analysis revealed that transcriptional and proteomics profiles at day 5 post-treatment were not affected by Peginterferon Lambda treatment (Figure 2D, 2G, and Supplemental Figure 1). We also tested the effect of Peginterferon Lambda treatment on each individual immune measure at day 5 post-treatment. We found that only two plasma proteins (HSD1B1 and LAMP3) that were significantly affected by Peginterferon Lambda treatment (Supplemental Figure 1F). However, regression analysis demonstrated that neither HSD1B1 nor LAMP3 were associated with patient outcomes, including disease progression, oropharyngeal viral load, SARS-CoV-2 specific T cell responses, or antibody responses measured at day 28. Furthermore, we found no significant differences in SARS-CoV-2 specific T cell responses (at day 28 after enrollment) and antibody responses (at day 28 and month 7 after enrollment) between the two treatment arms (Supplemental Figure 1), as reported previously^22^. Taken together, Peginterferon Lambda treatment did not show noticeable effects on the immune response in COVID-19 outpatients. Therefore, we combined the data from the control and treatment arms together for all downstream analysis.

### Trajectory analysis reveal sequential activation of immune modules in COVID-19 patients

**Figure 3:**
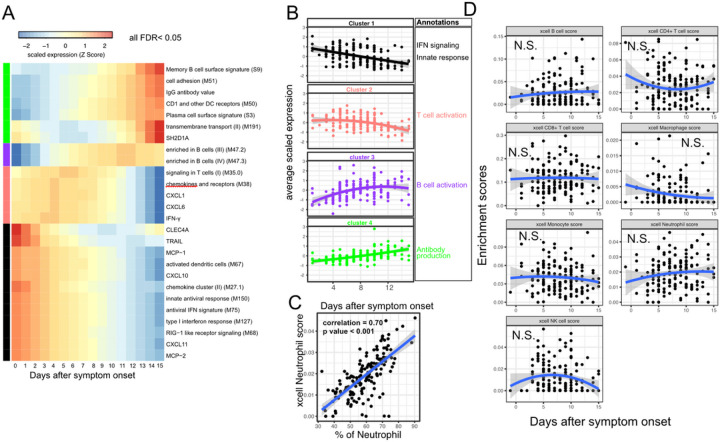
Trajectory analysis reveals sequential activation of immune modules in COVID-19 patients. (A) The fitted expression level of immune modules and plasma proteins at 0–15 days after symptom onset. The values are calculated by fitting quadratic regressions and are scaled to a mean of 0 and a standard deviation of 1. The color bar on the left side shows the clustering membership of the modules and plasma proteins. Modules and proteins with FDR<0.05 are shown. (B) the average trajectory of the clusters. We scaled the expression level of each module and plasma proteins to a mean of 0 and a standard deviation of 1. We then calculated the average scaled expression of all members in the clusters. Each dot represents the mean expression in each blood sample. The lines represent the fitted quadratic regression. The grey areas represent the 95% confidence intervals. (C) We estimated the spearman correlation between the neutrophil enrichment score using the xCell. The plot shows the correlation between the xCell score and the counted neutrophil percentage in whole blood. (D) The relationship between xCell enrichment score and days after symptom onset.

We next characterized the trajectory of early transcriptomic and proteomic responses using the RNAseq and Olink data as a function of time since symptom onset. To reduce the dimensionality and improve interpretability, we calculated the enrichment score of different immune modules (based on Blood Transcription Modules (BTM)^23^) from the RNAseq data. We then combined the enrichment scores and Olink measurements into a single dataset for the trajectory analysis. We fitted the data with quadratic regression to capture the non-linear dynamics of the modules and proteins. We identified 15 immune modules and 10 plasma proteins that varied as a function of time since symptom onset (False Discovery Rate (FDR) < 0.05, Figure 3A and Supplemental Table 2). Among them, 16 immune modules or proteins showed nonlinear dynamics, as indicated by significant coefficients of the quadratic term (Supplemental Table 2).

We performed clustering analysis and identified four clusters based on the trajectory of the significant modules and proteins (Figure 3A–B). Cluster 1 contains interferon-related modules and proteins known to be activated by interferon signaling, including MCP-1, MCP-2, CXCL10 and CXCL11^24–26^. The trajectories in cluster 1 already reached the peak at the time of symptom onset and monotonically decreased over time. The trajectories in cluster 2 peaked at 1–5 days after symptom onset and contain Interferon-γ and modules related to T cell activation. Interestingly, it also contains several myeloid cell attracting chemokines (CXCL1 and CXCL6) and the innate cell response modules. Cluster 3 peaked between 10 to 14 days after the symptom onset and is characterized by modules related to B cells. Cluster 4 trajectories monotonically increase after symptom onset and are characterized by the increasing S protein binding IgG level and related plasma B cell modules. The trajectory analysis revealed the sequential activation of interferon signaling, myeloid cells, Interferon-γ, T cells, B cell and antibody production within the first 15 days of symptom onset. Consistent with the BTM analysis, a pathway analysis using Gene Ontology identified a similar sequential activation of immune pathways within the first 15 days of symptom onset. (Supplemental Figure 2A).

To characterize how the composition of blood immune cells change over time, we used a previously established tool named xCell to estimate the enrichment score of the major immune cells^27^. As a positive control, we compared the neutrophil score with the neutrophil count data obtained from clinical lab tests and found high correlation between them (Figure 3C). Quadratic regression did not find significant associations between the major cell types and the time since symptom onset (Figure 3D). The results suggest that the trajectories of different immune modules (Figure 3A) are mainly driven by the activation of corresponding immune cells rather than the composition change of major immune cell types.

### Variations in early immune responses are associated with disease severity in COVID-19 patients.

We next sought to identify immune modules and plasma proteins associated with disease progression in COVID-19 outpatients. At the time of sample collection (day 0 and day 5 after enrollment), the majority of subjects showed either mild to moderate symptoms that subsequently resolved (n=92) or were asymptomatic (n=8). However, 8 patients with initially mild to moderate symptoms later developed progressive and more severe symptoms and presented to the emergency department or were hospitalized (median 2 days to progression, range 1–13 days; Supplementary Table 1). We defined these individuals as “progressors”, and used regression models to identify immune modules and plasma proteins to compare these participants with those who didn’t seek care at the hospital (non-progressors), while controlling for days after symptom onset.

**Figure 4:**
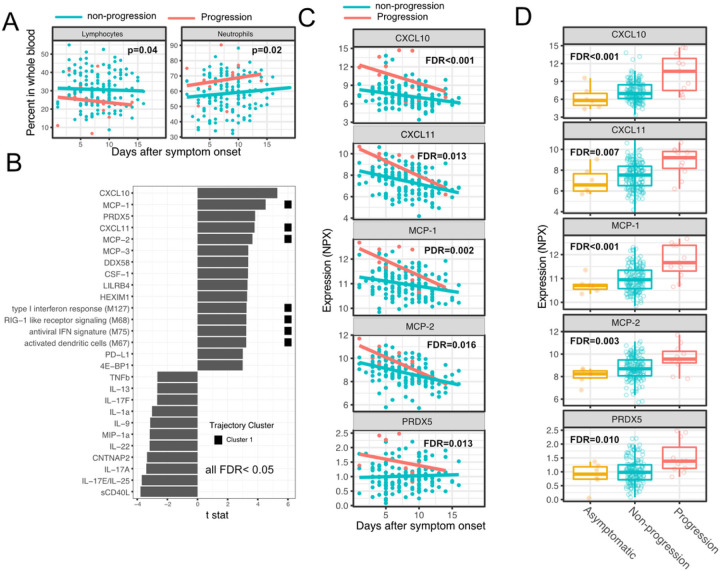
Variations in early immune responses are associated with disease severity in COVID-19 patients. (A) Scatter plot comparing the percentage of lymphocytes and neutrophils in whole blood between moderate and severe cases. The lines represent the fitted linear relationship between the percentages and the time after symptom onset. We fitted regression models to test the relationship between the immune measurements and disease progression while controlling for the time after symptom onset. The p values for the disease progression are reported. (B) We fitted regression models to test the relationship between the immune measurements and symptom severity while controlling for the time after symptom onset. The bar plot shows the t score of the regression coefficient for disease progression. The colored squares represent the clusters each immune measurement belongs to. The clusters are defined in Fig. 3A. FDR represent the p value of the regression coefficient of the disease progression term after multiple testing adjustment. (C) Scatter plot comparing the plasma protein levels between moderate and severe cases. The lines represent the fitted linear relationship between the percentages and the time after symptom onset. The top 5 significant proteins are shown. Data from asymptomatic cases are omitted, as their symptom onset time was unknown. FDR are the same as in B. (D) Box plots comparing the plasma protein levels between asymptomatic, non-progressed and progressed cases. FDR represent the p value from one way ANOVA after multiple testing adjustment.

As two positive controls, we confirmed well documented findings that lymphocyte percentages were negatively correlated with symptom severity and neutrophil percentages were positively correlated with symptom severity (Figure 4A)^28^. In addition, our regression analysis identified 4 immune modules and 23 plasma proteins that were significantly associated with symptom severity (FDR<0.05, Figure 4B–C, and Supplemental table 3). The proteins and modules from cluster 1 (as identified above in Figure 3A), including the Type I Interferon response, Rig-I signaling, and multiple proteins known to be induced by interferon signaling including CXCL10 (also known as Interferon Gamma-induced protein, IP-10), MCP-1, MCP-2 and CXCL11, were significantly enriched in individuals who experienced disease progression vs. non-progressors (Fisher’s exact test, p < 0.001). As our regression analysis excluded asymptomatic individuals, we performed one-way ANOVA analysis without adjusting for symptom onset time. The results from the ANOVA analysis were consistent with the regression analysis (Figure 4D). Pathway analysis using Gene Ontology identified similar Interferon and Rig-I related pathways to be associated with the symptom progression (Supplemental Figure 2B). Together, these data highlight an association between early innate immune activation and disease progression.

### Early proteomic and transcriptomic signatures show long-term association with virologic and immunologic outcomes.

**Figure 5:**
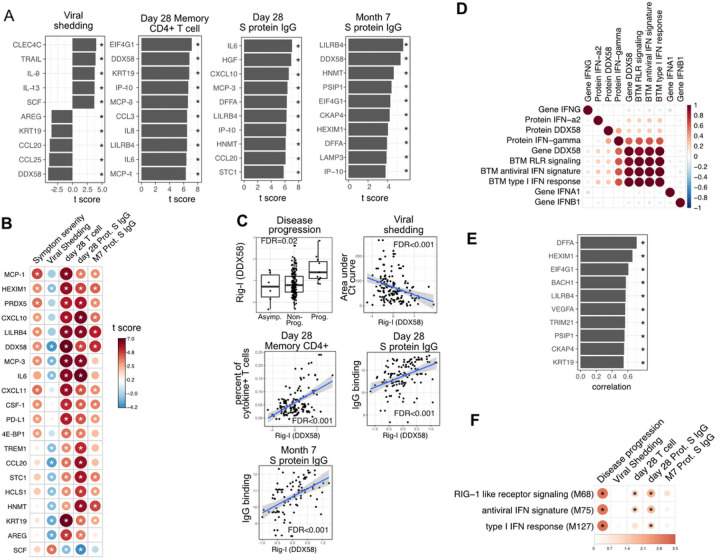
Plasma RIG-I is a biomarker for disease progression, viral shedding, T cell activity, and spike (S)-binding IgG levels (A) The association between plasma proteins and viral shedding, memory T cell activity, and anti-S binding IgG levels. Memory CD4+ T cell activities are measured by the percent of cytokine positive T cells (TNF-α+ or TNFγ+ or IL21+) after spike protein stimulation. T cells are collected from patients 28 days after enrollment. Spike protein-binding IgG levels are measured 28 days or 7 months after enrollment. We fitted regression models to test the relationship between the immune measurements and viral shedding, memory T cell activity, and S protein binding IgG level while controlling for the time after symptom onset. The bar plot shows the t score of the regression coefficient for viral shedding, memory T cell activity, and anti-S binding IgG levels. (B) Association between plasma proteins and multiple outcomes. The heatmaps include immune measurements that are significantly associated (indicated by stars) with at least 3 outcomes. (C) Correlation between plasma RIG-I (DDX58) and disease progression, viral shedding, memory T cell activity, and S protein binding IgG levels. (D) Correlation between plasma RIG-I protein and selected level of plasma proteins, genes, and BTM modules. (E) The top 10 plasma proteins correlated with plasma RIG-I protein. (F) The association between Rig-I and interferon related BTMs and the outcomes of COVID-19 patients. Stars represent FDR<0.05.

We next examined associations between plasma proteins measured early in the course of infection and oropharyngeal viral load (measured by the area under the Ct curve from day 0 to 14 post enrollment), SARS-CoV-2-specific T cells measured 28 days post-enrollment (Supplemental Figure 4; Supplemental Table 4), and anti-Spike binding antibodies measured at 28 days and 7 months post-enrollment. Importantly, these virologic and immunologic outcomes were not significantly associated with disease progression, suggesting that they are not driven by more severe disease (Supplemental Figure 5). We identified 36 plasma proteins significantly associated with oropharyngeal viral load (top 10 significant proteins shown in Figure 5A, Supplemental table 3). Higher levels of several of these proteins were inversely correlated with viral load, including the cytosolic RNA sensor RIG-I (gene symbol DDX58), chemokines (CCL20 and CCL25), and other proteins (Keratin 19 {KRT19}, amphiregulin{AREG}) previously shown to be upregulated in COVID-19 patients^14^. In contrast, higher levels of C-Type Lectin Domain Family 4 Member C (CLEC4C, expressed by plasmacytoid dendritic cells), and TNF-related apoptosis-inducing ligand (TRAIL), were associated with higher oropharyngeal viral loads.

We identified 87 plasma proteins that were significantly associated with SARS-CoV-2-specific T cell responses at day 28, and 91 and 13 plasma proteins significantly associated with S protein-binding IgG at day 28 days and month 7, respectively (top 10 significant proteins shown in Figure 5A, Supplemental table 3). Several proteins were associated with higher levels of SARS-CoV-2-specific T cells and the antibody response, including RIG-I (gene symbol DDX58), chemokines (CXCL11), and other proteins (KRT19, AREG) also associated with control of viral load (Figure 5A and B).

Altogether, we identified 20 plasma proteins that were correlated with three out of four aspects of the patient outcomes (Figure 5B). To ensure that there were not significant interactions between treatment arm, early immune markers, and patient outcomes, we estimated associations using data from the placebo arm of the lambda trial only, with similar results to the pooled analysis (Supplemental Figure 6). Interestingly, higher plasma levels of RIG-I (gene symbol DDX58) were significantly associated with all examined clinical, virologic and immunologic outcomes (Figure 5C), including disease progression, lower oropharyngeal viral load, increased SARS-CoV-2 specific T cell responses, and increased levels of S protein-binding IgG to SARS-CoV-2. Since RIG-I is a cytosolic PRR that, upon recognition of short viral double-stranded RNA during a viral infection, leads to upregulation of interferon signaling^29^, we explored associations between plasma RIG-I levels and related immune measurements, including the mRNA-level and protein-level expression of RIG-I and interferons, as well as RIG-I and interferon-related modules. We found that the plasma RIG-I levels were only modestly correlated with mRNA-level expression of RIG-I (correlation = 0.23, p value = 0.004, Figure 5D), as well as Rig-I signaling and Interferon related modules (Figure 5D). In contrast, we found a strong correlation between plasma levels of RIG-I and plasma levels of DFFA, an intracellular protein known to be involved in apoptosis (Figure 5H)^30,31^. In addition, many of the other plasma proteins correlated with plasma RIG-I levels were intracellular proteins (Figure 5E), suggesting that plasma RIG-I levels may be driven by both increased intracellular RIG-I expression, as well as a cell death process that releases intracellular protein into the plasma. Analysis of RNA-sequencing data also identified associations between Rig-I, interferon signaling, and cell death related modules and clinical and immunologic outcomes of patients (Figure 5F and Supplemental Figure 2C). As plasma levels of RIG-I were not significantly associated with time since symptom onset (Supplemental Table 2), these data suggest that plasma RIG-I levels might serve as a powerful and stable biomarker for predicting several clinical, viral and immunological outcomes in patients with COVID-19.

### Similar trajectories of immune responses induced by SARS-CoV-2 infection and COVID-19 mRNA vaccine.

**Figure 6:**
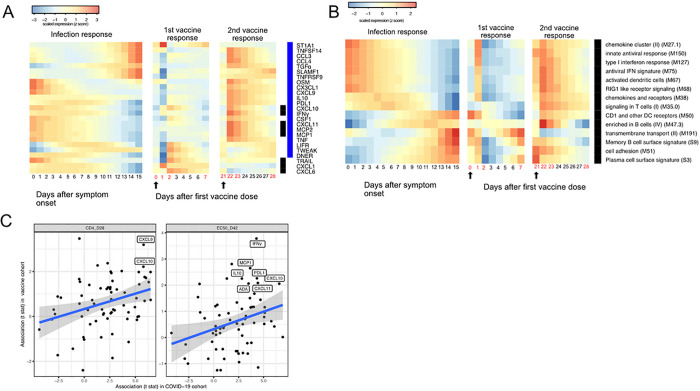
Comparing the immune response induced by SARS-CoV-2 infection and COVID-19 vaccine (BNT162b2). (A-B) The heat map shows the expression level of plasma proteins (A) and BTMs (B) at 0–15 days after symptom onset in COVID-19 patients (left), 0–7 days after the first does of vaccination and 21–28 days after the second dose of vaccination in healthy individuals (right). The values from the SARS-CoV-2 dataset are calculated by fitting quadratic regressions and are scaled to a mean of 0 and a standard deviation of 1. The values from the vaccination dataset are computed by fitting using linear interpolation between the measured time points (days in red color) and are scaled to a mean of 0 and a standard deviation of 1. The black bar on the right side shows the protein markers that are significantly associated with time in COVID-19 patients (FDR<0.05). The blue bar on the right side shows the protein markers that are significantly associated with time after vaccination (FDR<0.05). The black arrows indicate the time of the first and the second doses of vaccination. (C) Comparing the biomarkers of immune outcomes in COVID-19 dataset and the BNT162b2 dataset. We fitted regression models to test the association between the highest level of protein markers after first vaccination (day 0 - day 21) and the T cell (left) and antibody responses (Right). The t statistics from the regression model is compared with the t statistics from the COVID-19 dataset.

The BNT162b2 (Pfizer–BioNTech) vaccine has been widely used throughout the world and is highly effective in preventing SARS-CoV-2 infection, as well as protecting patients from severe symptoms after infections^19^. We leveraged a recently published dataset from a BNT162b2 vaccine study to compare the immune response induced by COVID-19 vaccine and SARS-CoV-2 infections^32^. Comparison of the datasets reveals that the immune response after the first dose of vaccination (day 0 to day 21) largely mirrors the trajectory of immune response after SARS-CoV-2 infection. Early proteins and BTMs in the SARS-CoV-2 infection dataset, including IFNγ, MCP1, CXCL11, MCP2, CXCL10 and interferon related transcriptional modules are upregulated within the first 7 days of the vaccination. Late immune markers in the SARS-CoV-2 infection dataset, including SLAMF1, TNFRSF9, CCL3, CCL4, TGFα, TNFSF14 and B cell related transcriptional modules are upregulated much later and show highest levels 21 days after the vaccination (Figure 6A and B). In contrast, the response after the second dose of vaccine (day 22 to day 28) is characterized by fast upregulation of both early and late immune measurements (Figure 6A–B). Interestingly, three proteins that are significantly upregulated in SARS-CoV-2 patients were not induced after the second dose of vaccine, including TRAIL, CXCL1 and CXCL6. All three proteins are highly expressed in neutrophils^33^, and have been shown to regulate neutrophil recruitment (CXCL1 and CXCL6) or apoptosis (TRAIL) during inflammation^34–36^. The lack of TRAIL, CXCL1 and CXCL6 suggests an absence of neutrophil response to the second dose of vaccine.

We next examined associations between plasma proteins and immunologic outcomes of BNT162b2 vaccination. The associations identified in the vaccine dataset are largely consistent with the associations identified in the COVID-19 infection dataset (Figure 6C). In particular, proteins that are associated with the T cell (CXCL9 and CXCL10) and antibody responses (INF-γ, MCP1, L10, PDL1, CXCL10, ADA and CXCL11) after infection were also associated with the T cell and antibody responses after vaccination, highlighting important similarities between infection-induced and vaccine-induced immunity. Furthermore, these results suggest that these plasma biomarkers may be useful correlates of protective immunity following both natural infection and vaccination.

### Plasma proteins predict symptom severity, T cell response and Spike protein-binding IgG levels in COVID-19 patients.

**Figure 7:**
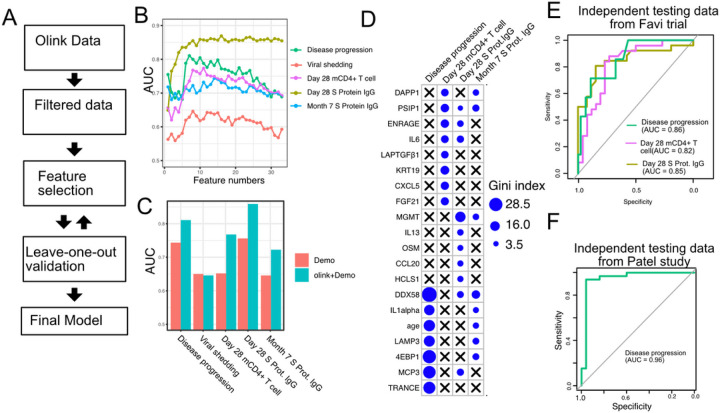
Plasma protein markers predict disease progression, T cell response, and S protein-binding IgG level in COVID-19 patients. (A) machine-learning procedure for predicting COVID-19 patient outcomes using Olink proteomics data. (B) Random forest models were built to predict symptom severity, S protein-binding IgG level at 28 days and 7 months after enrollment, and cytokine+ memory CD4+ T cells 28 days after enrollment. The plot shows the leave-one-out cross-validation performance (measured by AUC of the receiver operator characteristics curve (ROC)) achieved by random forest models with different numbers of features. (C) The leave-one-out cross-validation performance of the best performing models and the models using demographical data (age and sex) only. (D) Feature importance of the final random forest models for predicting symptom severity, S protein-binding IgG level at 28 days and 7 months after enrollment, and cytokine+ memory CD4+ T cells at 28 days after enrollment. (E) We generated an independent dataset using samples obtained from 64 COVID-19 patients enrolled in the placebo arm of a clinical trial of Favipiravir. The performance of the machine learning models was tested using the new dataset. (F) We used the final model to predict severe cases in an independent dataset and measured the performance of the model measured by the ROC curve.

Finally, we tested if plasma proteins measured early following infection can accurately predict disease progression, oropharyngeal viral load, and SARS-CoV-2 specific memory T cell and antibody responses manifested later in the study. We adopted a computation pipeline to select a small subset of predictive biomarkers from the 184 proteins measured by Olink assays. We used a leave-one-out cross-validation strategy to iteratively evaluate model performance, and Random Forest for feature selection and for building the final model (Figure 7A). Based on results from cross-validation, we selected between 7 to 10 protein markers measured at early infection to predict each of the five outcomes. The final models achieved cross-validation AUC of 0.82, 0.64, 0.77, 0.86 and 0.72 for predicting disease progression, oropharyngeal viral load, day 28 SARS-CoV-2 specific CD4+ T cell responses, day 28 spike protein binding IgG levels and month 7 spike protein binding IgG levels, respectively (Figure 7B).

We compared the final models to baseline models that use only demographic (age and gender) data. The selected protein markers substantially improved the prediction of disease progression, spike protein binding IgG levels at day 28 and month 7, and SARS-CoV-2 specific CD4+ T cell responses at day 28. On the other hand, protein markers did not improve the prediction for oropharyngeal viral load.

To validate the single-variable associations (Figure 5) and the multi-variable machine learning models (Figure 7A–D), we generated an independent dataset using longitudinal, acute, and convalescent samples obtained from 54 COVID-19 participants enrolled in the placebo arm of an outpatient clinical trial of Favipiravir (NCT04358549). Similar to participants in the Lambda trial, participants in the Favipiravir trial were recruited if they presented with initially mild to moderate COVID-19 at diagnosis, and the median duration of symptoms prior to randomization was 5 days, (Supplementary Table 5). Among study participants, 7/54 (13%) later developed progressive and more severe symptoms and presented to the emergency department or were hospitalized. We measured plasma proteomics by Olink at the time of enrollment (Day 0), and neutralizing antibody levels and SARS-CoV-2- specific CD4+ T cell responses 28 days post-enrollment. Using this new dataset, we validated associations between early proteomic markers and longitudinal clinical and immunology outcomes (Supplemental Figure 7). Importantly, we also demonstrate that machine learning models using 7–10 plasma protein markers measured during acute infection and developed from the Lambda dataset can accurately predict disease progression (AUC 0.86), SARS-CoV-2 specific CD4+ T cell responses (AUC 0.82), and the magnitude of Spike protein binding IgG (AUC 0.85) at 28 days post enrollment in the independent favipiravir dataset (Figure 7E).

We also tested if our model can accurately predict disease severity in a second independent dataset, and identified a published dataset that characterized plasma proteins from 58 COVID-19 patients (26 moderate cases and 34 severe cases)^37^. Our model was able to accurately identify severe cases in the independent dataset, achieving an AUC of 0.96. The individuals in the test dataset already manifested severe symptoms while our training dataset was collected before the onset of severe symptoms, potentially explaining the higher model performance in the test dataset than in the training dataset.

## Discussion

In this study, we longitudinally characterized the early immune response in patients who initially presented with mild to moderate COVID-19. With transcriptomic and proteomic profiling, we reveal a sequential activation of interferon signaling, T cells and B cells within 2 weeks of symptom onset. We also identified associations between early immune profiles and later clinical, virologic, and immunologic outcomes. In particular, plasma RIG-I levels, early interferon signaling, and related cytokines (CXCL10, MCP1, MCP-2 and MCP-3) were associated with multiple patient outcomes, including disease progression, viral shedding, and the SARS-CoV-2 specific T cell and antibody response measured up to 7 months after enrollment. We observed that the immune response after the first dose of SARS-CoV-2 mRNA vaccination largely recapitulates the trajectory of immune response after SARS-CoV-2 infection, and associations between early proteomic signatures and adaptive immune responses were similar following natural infection and vaccination. Finally, we demonstrate that machine learning models using 7–10 plasma protein markers are able to accurately predict disease progression, SARS-CoV-2 specific T cell magnitude, and the SARS-CoV-2 antibody response in independent datasets.

We found that variations in the early immune response following natural infection shape the long-term outcome of COVID-19 outpatients. In particular, early transcriptomic signatures of Type I IFN and RIG-I signaling, as well as elevated levels of downstream, interferon induced chemokines (CXCL10, CXCL11), were associated with an increased risk of subsequent disease progression. These results are consistent with some previously reported studies^10,38^, but not others, which found that severe disease is associated with a defective interferon response^6,39^. Some of these differences may be related to the timing of the assessment. Samples in our study were obtained in outpatients, prior to disease progression and hospitalization; in most other studies, patient samples were obtained at the time of hospitalization and/or when patients already had evidence of severe disease. This suggests a complex, non-monotonic relationship between the interferon response and disease severity.

Importantly, plasma levels of RIG-I, a protein not associated with time since symptom onset, was associated with all measured patient outcomes, including disease progression, oropharyngeal viral load, and SARS-CoV-2 specific T cell and antibody responses. RIG-I has been shown to be critically important in the response to several RNA viruses, including influenza virus, typically via interactions with the adapter protein mitochondrial antiviral-signaling protein (MAVS) and downstream Type I and Type III interferon upregulation. RIG-I was recently shown to play an important role in both sensing SARS-CoV-2 RNA and inhibiting SARS-CoV-2 replication in human lung cells, but not via downstream MAVS induction^40^. Rather, interactions between the RIG-I helicase domain and SARS-CoV-2 RNA induced an inhibitory effect on viral replication, independent of downstream interferon upregulation^40^. Our data showing an inverse association between plasma RIG-I levels and viral load are consistent with RIG-I having an important role in restricting early virus replication. As RIG-I is an intracellular RNA sensor, we sought to better understand why levels of RIG-I were elevated in the plasma by assessing associations with other transcriptomic modules and plasma proteins. Plasma RIG-I levels were modestly correlated with mRNA-level expression of RIG-I and RIG-I signaling modules, and strongly correlated with plasma levels of several intracellular proteins, including DFFA, an intracellular protein known to be involved in cell death^31^, suggesting that plasma RIG-I levels may reflect both increased gene expression and increased cellular apoptosis. This hypothesis is consistent with a recent report which observed significant associations between gene expression signatures of apoptosis in plasmacytoid dendritic cells with increased disease severity^9^.

We also observed that higher expression of three CCR2 ligands MCP1, MCP2 and MPC3 were associated with disease progression. Higher plasma MCP3 levels have previously been shown to be elevated in SARS-CoV-2 infected patients with severe disease in comparison with those without^38^, and transcriptome-wide association in lung tissue has found that higher expression of monocyte-macrophage chemotactic receptor (CCR2), the receptor for MCP1 and MCP3, is associated with severe COVID-19^41^. However, we also observed that these CCR2 ligands are associated with positive virologic and immunologic outcomes, including reduced oropharyngeal viral load, and increased SARS-CoV-2 specific T cells and S protein-binding IgG levels. Consistent with a potential beneficial role, murine studies have found that CCR2 is essential for the survival of mice after pathogen challenge^42–44^. Taken together, these results demonstrated the complex role of CCR2 signaling in regulating immune responses. While essential for an effective immune response, overexpression may lead to severe symptoms and tissue damage. Therapeutic strategies to balance the positive and negative effects of CCR2 signaling may benefit the management of COVID-19 patients.

Our study design allowed us to compare immune responses following Peginterferon Lambda treatment. Although there is literature that suggests Interferon Lambda-1a (IL-29) may impact immune function *in vitro* and in animal models^45^, the impact of therapeutic administration on immune function in humans remains unclear. In this study, we found that administration of Peginterferon Lambda had no discernible effect on the immunologic profiles of participants. Specifically, we observed no significant impact of Lambda on whole blood transcriptomic or proteomic profiles 5 days post-administration, nor in SARS-CoV-2 specific T cell and antibody responses 28 days post-administration. There are several possible reasons for these findings. First, the dosing used in this trial may not have achieved therapeutic levels in the upper respiratory epithelia, where its impact on immune cells might be expected to occur^21^. Second, the median symptom duration was 5 days at the time of randomization, and 40% of participants were already SARS-CoV-2 IgG positive at enrollment. It is possible that earlier administration, or prophylactic administration prior to established infection, may have had a different impact on immune outcomes. Arguing against this, in sensitivity analyses, we observed no difference in the treatment effect on immune profiles among individuals who were SARS-CoV-2 seronegative at enrollment.

Although naturally acquired SARS-CoV-2 infection results in protective antibody and T cell immune responses, reinfections can occur, and the precise determinants driving susceptibility to reinfection remain unclear^46^. The BNT162b2 (Pfizer–BioNTech) vaccine has been shown to be highly effective in preventing SARS-CoV-2 infection^19^, although breakthrough cases have been increasingly reported since its approval^47^. Comparing the vaccine response with the immune response of natural infection may shed light on determinants of protective immunity to SARS-CoV-2, and potential ways to improve COVID-19 vaccines. Our analysis reveals that the proteomic response of the BNT162b2 vaccine mirrors in many ways the proteomic response after SARS-CoV-2 infection. Furthermore, several associations between early protein markers and protective immunity (T cell and antibody responses) were shared between COVID-19 patients and individuals who received the BNT162b2 vaccine. These results suggest that plasma biomarkers might be useful to identify individuals at risk of SARS-CoV2 reinfection as well as breakthrough infection after vaccination.

Our study has some limitations. First, while we identified multiple associations between early immune measures and the outcome of COVID-19 patients, we did not establish causal relationships between them. Future studies are needed to perturb key immune modules in the early immune response and test their effect on the patient outcomes. Second, our study measured the immune response during the first 2 weeks after symptom onset in COVID-19 patients. Earlier immune responses between the initial infection and symptom onset have not been characterized. This is due to the difficulty to detect pre-symptomatic COVID-19 infection. Routine SARS-COV-2 monitoring in a select cohort will be required to acquire samples prior to and immediately after the infection in order to assess whether pre-infection signatures predict outcomes in COVID-19 patients. In addition, our trajectory analysis is based on the population-level data with only two timepoints sampled per patient. Further studies with more frequent sampling will be needed to confirm the immune trajectory at the individual level. Finally, we used median values as cutoffs to identify individuals with high or low T cell and antibody responses when training the machine learning model. However, the use of median cutoff is not ideal, as it does not reflect the minimal threshold of protective immunity. Further studies are needed to identify clinically relevant thresholds and to test the machine learning models for predicting protection against re-infection.

Identification of patients at high risk of disease progression remains a critical need in management of patients with COVID-19. Several novel therapeutics have recently been issued emergency use authorization by the Federal Drug Administration, including monoclonal antibodies (e.g., casirivimab and imdevimab, sotrovimab), nucleoside inhibitors (molnupiravir), and protease inhibitors (nirmaltrevir/ritonavir). These therapeutics have been shown to be effective in reducing the risk of hospitalization or death among patients with high risk of disease progression in outpatient studies, but their supplies remain limited. Our data suggest that measurement of plasma proteins at the time of diagnosis could be a powerful adjunct to identify those patients who would most benefit from therapeutic interventions aimed at preventing progressive disease and hospitalization. Furthermore, our models can potentially be used to predict the degree of immune memory variation following natural infection and vaccination.

## Methods

### Lambda Study Design and Oversight

Data and samples were obtained from a Phase 2, single-blind, randomized placebo-controlled trial to evaluate the efficacy of Lambda in reducing the duration of viral shedding in outpatients. The trial was conducted within the Stanford Health Care System. Adults aged 18–65 years with an FDA emergency use authorized reverse transcription-polymerase chain reaction (RT-PCR) positive for SARS-CoV-2 within 72 hours from swab to the time of enrollment were eligible for participation in this study. We included both symptomatic and asymptomatic patients based on the previous finding that the detected infectious virus were similar in samples from asymptomatic and symptomatic persons^47^. Symptomatic individuals were eligible given the presence of mild to moderate symptoms without signs of respiratory distress. Asymptomatic individuals were eligible if infection was the initial diagnosis of SARS-CoV-2 infection. Exclusion criteria included current or imminent hospitalization, respiratory rate >20 breaths per minute, room air oxygen saturation <94%, pregnancy or breastfeeding, history of decompensated liver disease, recent use of interferons, antibiotics, anticoagulants or other investigational and/or immunomodulatory agents for treatment of COVID-19, and prespecified lab abnormalities. Full eligibility criteria are provided in the study protocol. The protocol was amended on June 16^th^, 2020 after 54 participants were enrolled but before results were available to include adults up to 75 years of age and eliminate exclusion criteria for low white blood cell and lymphocyte count. The trial was registered at ClinicalTrials.gov (NCT04331899). The study was performed as an investigator-initiated clinical trial with the FDA (IND 419217), and approved by the Institutional Review Board of Stanford University.

### Participant Follow Up and Sample Collection

Participants completed a daily symptom questionnaire using REDCap Cloud version 1.5. In-person follow-up visits were conducted at day 1, 3, 5, 7, 10, 14, 21, and 28, with assessment of symptoms and vitals, and collection of oropharyngeal swabs (FLOQ Swabs; Copan Diagnostics). Peripheral blood was collected at enrollment, day 5, day 28, and month 7 post randomization. Whole blood was collected in Paxgene Tubes, and remaining blood was processed for plasma and peripheral blood mononuclear cells.

### Clinical Laboratory procedures

Laboratory measurements were performed by trained study personnel using point-of-care CLIA-waived devices or in the Stanford Health Care Clinical Laboratory. Oropharyngeal swabs were tested for SARS-CoV-2 in the Stanford Clinical Virology Laboratory using an emergency use authorized, laboratory-developed, RT-PCR. Centers for Disease Control and Prevention guidelines identify oropharyngeal swabs as acceptable upper respiratory specimens to test for the presence of SARS-CoV-2 RNA, and detection of SARS-CoV-2 RNA swabs using oropharyngeal swabs was analytically validated in the Stanford virology laboratory.

### Whole blood transcriptomics

Whole blood transcriptomics were performed at Novogene Corporation, Inc. Briefly, whole blood samples collected in Paxgene Tubes were first treated with Proteinase K, and then RNA extraction performed using Quick-RNA MagBead Kit (R2132) on KingFisher followed by sample quality control checks using a Qubit and Bioanalyzer 2100. Libraries were prepared using ZymoSeq RiboFree Total RNA Library Kit (R3000). Sequencing took place on a Nova6000 on an S4 lane, 30M paired reads, PE 150.

### Whole blood transcriptomic data analysis

The transcript-level count data and transcript per million (TPM) data was calculated using Kallisto^48^ (v0.46.2) and human cDNA index produced using kallisto on Ensembl v96 transcriptomes. For each RNA-seq sample, we calculated the single-sample enrichment score of the blood transcription modules (BTM) using the fgsea R package^49^. The enrichment scores of the BTMs were normally distributed across samples and are treated as variables, similar to individual protein markers, in the downstream analysis.

### Plasma protein profiling using Olink panels

We measured proteins in plasma using Olink multiplex proximity extension assay (PEA) inflammation panel and immune response panel (Olink proteomics, www.olink.com) according to the manufacturer’s instructions. The PEA is a dual-recognition immunoassay, where two matched antibodies labeled with unique DNA oligonucleotides simultaneously bind to a target protein in solution. This brings the two antibodies into proximity, allowing their DNA oligonucleotides to hybridize, serving as a template for a DNA polymerase-dependent extension step. This creates a double-stranded DNA “barcode” unique for the specific antigen and quantitatively proportional to the initial concentration of target protein. The hybridization and extension are immediately followed by PCR amplification and the amplicon is then finally quantified by microfluidic qPCR.

### T Cell Assays

SARS-CoV-2 specific T cell peptide pools were purchased from Miltenyi Biotec (PepTivator® SARS-CoV-2 Prot_S, Prot_S1, Prot N, and Prot M) and resuspended in DMSO. These PepTivator® reagents are pools of lyophilized peptides of 15 amino acid length with 11 amino acid overlap, covering immunodominant sequence domains of the Spike (S and S1) (aa sequence 1–1273), Nucleocapsid (N) or Membrane (M) proteins of SARS-CoV-2.

Antigen-specific T cell responses were measured using an intracellular cytokine staining assay. Briefly, cryopreserved PBMCs were thawed, counted, and resuspended in complete RPMI (RPMI (Corning) supplemented with 10% FBS (Gibco), 100 IU Penicillin (Corning), 100 ug/ml Streptomycin (Corning), 1 mM Hepes (Corning) and 2 mM L-glutamine (Corning)). The cells were plated in 96-well U bottom plates at 1×10e6 PBMCs per well and then rested overnight at 37°C in a CO2 incubator. The following morning, cells were cultured in presence of either SARS-CoV-2 peptides (1 μg/ml), PMA (300 ng/ml) and Ionomycin (1.5 μg/ml) as positive control, or media as a negative control for 6 hours at 37°C. All conditions were in the presence of brefeldin A (BD Pharmingen), monensin (BD Pharmingen), and CD107a. After a 6-hour incubation, cells were washed and surface stained for CCR7 for 15 min at 37°C, followed by the remaining surface stain for 30 min at room temperature (RT) in the dark. Thereafter, cells were washed twice with PBS containing 0.5% BSA and 2 mM EDTA, then fixed/permeabilized (FIX & PERM® Cell Permeabilization Kit, Invitrogen) and stained with intracellular antibodies for 20 min at RT in the dark. A complete list of antibodies is listed in Supplementary Methods. All samples were analyzed on an Attune NXT flow cytometer and analyzed with FlowJo X (Tree Star) software.

### Antibody Assays

IgG antibody titers against the SARS-CoV-2 full length spike protein were assessed by enzyme-linked immunosorbent assay (ELISA)27. Briefly, 96 Well Half-Area microplates (Corning (Millipore Sigma)) plates were coated with antigens at 2 μg/ml in PBS for 1h at RT. Next, the plates were blocked for an hour with 3% non-fat milk in PBS with 0.1% Tween 20 (PBST). Plasma was diluted fivefold starting at 1:50 in 1% non-fat milk in PBST. 25 μl of the diluted plasma was added to each well and incubated for 2h at RT. Following primary incubation, 25 μl of 1:5000 diluted horse radish peroxidase (HRP) conjugated anti-Human IgG secondary antibodies (Southern Biotech) were added and incubated for 1h at RT. The plates were developed by adding 25 μl/well of the chromogenic substrate 3,3′,5,5′-tetramethylbenzidine (TMB) solution (Millipore Sigma). The reaction was stopped with 0.2N sulphuric acid (Sigma) and absorbance was measured at 450nm (iD5 SPECTRAmax, Molecular Devices). The plates were washed 5 times with PBST between each step and an additional wash with PBS was done before developing the plates. All data were normalized between the same positive and negative controls and the binding AUC were calculated using GraphPad PRISM (Version 9).

### Quantifying oropharyngeal viral load

We identified the cycle threshold (Ct) value using the fluorescence vs cycle data reported from RT-PCR scanner. We subtract the Ct value from the detect limit (Ct=41) to quantify the viral shedding in each OP swap. We plotted the viral shedding in each visit versus time and calculated the area under the curve using numerical integration based on the trapezoid rule.

### Analysis of Olink data from the vaccine study

The olink data from the mRNA vaccine study was previously obtained and published ^30^.We tested if the level of the proteins are significantly altered after vaccination using ANOVA (expression ~ time), where time is treated as a categorical variable to account for non-linear behavior of the proteins. P values from the ANOVA models are adjusted using the False Discovery Rate (FDR) method^50^. To visualize the trajectory of the proteins, we imputed the protein level in each day using linear interpolation with the ‘approx’ function in R.

### Statistical analysis

Principal component analysis was conducted by applying the prcomp function in base R to the whole Olink dataset or the top 500 genes with the highest variance. To access the association between the principal components and clinical data, we fitted regression models (PC ~ clinical variable). The percent of variances explained by the clinical variable is used to measure the association.

We accessed the association between the expression of immune modules or protein markers with time using the regression model (expression ~ time + time^2^). Goodness of fit analysis using BIC shows that polynomial terms with orders higher than 2 do not improve the model (Supplemental Figure 3). It should be noted that our study contains repeated measures of the same individuals in two time points (0 and 5 days after enrollment). While including subjects as random effects in the regression model allows the model to adjust for individual differences, it resulted in near-singular fits of the data for many of the immune measurements. To avoid model over-fitting, we decided to only include the fixed effects (time) in our model. To find significant associations, we compared the model with the base model (expression ~ 1) and used the F test to calculate the p value. We adjusted the p value using the False Discovery Rate (FDR) method. We performed a parallel analysis using mixed-effect models [expression ~ time + time^2^ + subject ID (random effect)] to fit the data and found that all significant (FDR<0.05) variables identified using the fixed effect model were also significant in the mixed-effect model (Supplemental Table 2).

We estimated the enrichment score of the major immune cell types using the xCell package^27^. The association between the xCell scores and time were tested using the same regression method described above.

We tested the association between immune measurements and symptom severity using regression models (measurements ~ symptom severity + time + time^2^) and the lm function in R. The p value of the symptom severity term is adjusted using the FDR method. Similar regression models were used to test the association between immune measurements and other outcomes, including the oropharyngeal viral load, the memory CD4+ T cell activity and S protein binding IgG levels. To test between immune measurements and symptom severity without adjusting the time to symptom onset, we performed a one-way anova analysis using the lm function in R (measurements ~ symptom severity).

To estimate the time in which an immune measurement reaches the maximum level, we first fit a quadratic regression model (measurement ~ time + time^2^). We then identified the day (between 0 – 15 after symptom onset) in which the fitted regression model reached the maxim. We repeated the process 100 times to estimate the variance of the time.

### Predictive modeling

We used the protein measurements (measured by Olink assays) to predict the clinical, virological and memory T cell activity and IgG antibodies. Since the outcomes are a mixture of categorical (symptom severity) and continuous (viral load, T cell and antibody responses) variables, we framed all prediction tasks as classification problems by dichotomizing the continuous variables using median as cutoffs. To prevent overfitting, we selected 30 proteins with the highest variance as input data, as the highly variable proteins best capture the inter-subject difference across the COVID-19 patients. We further select features using random forest and leave-one-out validation. In step 1, we train a random forest model using data from all samples, but one left out sample. In step 2, we rank the feature importance of the 30 protein markers based on the gini index reported by the random forest model. In step 3, We train reduced random forest models with 1–29 most important proteins. In step 4, we predict the outcomes using the data from the left-out sample. We repeat steps 1–4 until we predict the outcome of all samples. We calculate the model performance using the area under the receiver operator characteristic curve (AUC). The variable combinations that give rise to the highest AUC are selected as the optimal model. The optimal model for predicting symptom severity was tested using Olink data from two independent studies.

### Data availability

The RNA-sequencing data is available in GEO under the accession number GSE178967 *(reviewer token: ypodeiiyphsvvyr)*. The Olink, clinical, virological, and immunological, as well as the machine learning models, are available in the github repository:

(https://github.com/hzc363/COVID19_system_immunology).

Note to reviewers: the github is currently private, but the data can be viewed at:

https://stanfordmedicine.box.com/s/humi9rlqqofl6pz1tqmf60rszui2aauy

All codes for data analysis are available upon request.

## Supplementary Material

Supplement 1

Supplement 2

Supplement 3
