# Supplementary material for: Early immune responses have long-term associations with clinical, virologic, and immunologic outcomes in patients with COVID-19
Source: Res Sq. 2022 Feb 2:rs.3.rs-847082. Preprint. [Version 1] doi: 10.21203/rs.3.rs-847082/v1 (PMC8820672; doi:10.21203/rs.3.rs-847082/v1)
Supplement: Supplement 3 [file b5b16ae3fbb9591aefdcfda1.docx]

**Supplementary Material:**

Early immune responses have long-term associations with clinical, virologic, and immunologic outcomes in patients with COVID-19

Zicheng Hu^1,2*^, Kattria van der Ploeg^3^, Saborni Chakraborty^3^, Prabhu Arunachalam^4^, Diego Martinez Mori^3^, Karen B. Jacobson^3^, Hector Bonilla^3^, Julie Parsonnet^3,5^, Jason Andrews^3^, Marisa Holubar^3^, Aruna Subramanian^3^, Chaitan Khosla^6^, Yvonne Maldonado^7^, Haley Hedlin^8^, Lauren de la Parte^3^, Kathleen Dantzler^3^, Maureen Ty^3^, Gene S Tan^9^, Catherine A. Blish^3,10^, Saki Takahashi^11^, Isabel Rodriguez-Barraquer^11^, Bryan Greenhouse^10,11^, Atul J. Butte^1^, Upinder Singh^3,12^, Bali Pulendran^4,11,12^, Taia T. Wang^3,10,12^, Prasanna Jagannathan^3,10*^

^1^ Bakar Computational Health Sciences Institute, University of California, San Francisco

^2^ Department of Microbiology and Immunology, University of California, San Francisco

^3^ Department of Medicine, Stanford University

^4^ Institute for Immunity, Transplantation, and Infection, Stanford University, Stanford, CA, USA

^5^ Department of Epidemiology and Population Health, Stanford University, Stanford CA, USA

^6^ ChEM-H, Stanford University, Stanford, CA USA

^7^ Department of Pediatrics, Stanford University, Stanford, CA, USA

^8^ Quantitative Sciences Unit, Stanford University, Stanford, CA, USA

^9^ J. Craig Venter Institute, San Diego, USA

^10^ Chan Zuckerberg Biohub, San Francisco, CA

^11^ Department of Medicine, University of California, San Francisco

^12^ Department of Microbiology and Immunology, Stanford University

^13^ Department of Pathology, Stanford University, Stanford CA, USA

*Corresponding authors

## Supplemental Table 1: Characteristics of Study Participants (Lambda study)

|  | **Treatment arm** | |  |
| --- | --- | --- | --- |
|  | **Lambda (N=54)** | **Placebo (N=54)** | **Overall (N=108)** |
| **Age in years**, median (range) | 37 (18-65) | 36 (20-71) | 37 (18-71) |
| **Male**, n (%) | 32 (59.3%) | 30 (55.6%) | 62 (57.4%) |
| **Race / Ethnicity**, n (%) |  |  |  |
| Latinx | 30 (56.7%) | 37 (68.3%) | 67 (62.0%) |
| White | 18 (30.0%) | 12 (25.0%) | 30 (27.8%) |
| Asian | 4 (5.0%) | 5 (6.7%) | 9 (8.3%) |
| Native Hawaiian or other Pacific Islander | 2 (3.3%) | 0 (0%) | 2 (1.9%) |
| **BMI (kg/m^2^)**, median (IQR) | 27.6 (25.4-31.1) | 28.5 (24.8-32.3) | 27.7 (24.9-32.0) |
| **Comorbid conditions** |  |  |  |
| Hypertension | 9 (16.7%) | 5 (6.8%) | 14 (13.0%) |
| Diabetes | 4 (7.4%) | 8 (14.8%) | 12 (11.1%) |
| Asthma | 2 (3.7%) | 2 (3.7%) | 4 (3.7%) |
| Heart Disease | 3 (4.1%) | 1 (1.9%) | 4 (3.7%) |
| **Asymptomatic at baseline**, n (%) | 5 (9.3%) | 3 (5.6%) | 8 (7.4%) |
| **Duration of symptoms in days prior to randomization**, median (IQR) ^1^ | 4 (3-6) | 5 (3-5) | 5 (3-6) |
| **Baseline oropharyngeal SARS-CoV-2 cycle threshold, median (IQR) ^2^** | 30.9 (26.4-33.8) | 29.3 (26.4-34.3) | 30.3 (26.4-34.3) |
| **Baseline SARS-CoV-2 IgG seropositivity**, n (%) | 17 (31.5%) | 24 (44.4%) | 41 (38.0%) |
| **Outcomes after Randomization** |  |  |  |
| Days until viral shedding cessation, median  (IQR) | 7 (5-13) | 7 (5-10) | 7 (5-13) |
| Hospitalization by day 28, n (%) | 2 (3.7%) | 2 (3.7%) | 4 (3.7%) |
| Hospitalization and/or emergency room  visit by day 28, n (%) | 5 (9.3%) | 3 (5.6%) | 8 (7.4%) |

##### **Supplemental Table 4: ICS antibody panel**

| **ICS Antibody Panel** | | | | | |
| --- | --- | --- | --- | --- | --- |
| **Surface Antibodies** | **Fluorochrome** | **Clone** | **Vendor** | **Catalog** | **Amount Per 50 uL** |
| CCR7 | BV421 | G043H7 | BioLegend | 353208 | 2.5uL |
| CD14  CD19   LIVE/DEAD | BV510  BV510  Aqua | M5E2  HB19 | BioLegend  BioLegend  Invitrogen | 301842  302242  L34965 | 0.5uL  0.5 uL  0.25uL |
| CD45RA | BV605 | HI100 | BioLegend | 304134 | 0.4uL |
| CD4 | BV650 | RPA-T4 | BioLegend | 300536 | 1uL |
| CD8A | BV785 | RPA-T8 | BioLegend | 301046 | 1uL |
| CD107A | FITC | H4A3 | BioLegend | 328606 | 1uL |
| CD3 | APC-H7 | SK7 | BD | 560176 | 2.5uL |
|  |  |  |  |  |  |
| **Intracellular Antibodies** | **Fluorochrome** | **Clone** | **Vendor** | **Catalog** | **Amount Per 50 uL** |
| IFN-g | PerCP Cy5.5 | 4S.B3 | BioLegend | 502526 | 0.5uL |
| IL-21 | eFluor660 | eBio3A3-N2 | eBioscience | 50-7219-42 | 1.25uL |
| TNF | AF700 | MAb11 | BD | 557996 | 0.5uL |

## Supplemental Table 5: Characteristics of Validation Study Participants (Placebo arm, Favipiravir trial)

| **Characteristic** | **N=54** |
| --- | --- |
| **Age in years**, median (range) | 44 (20-73) |
| **Male**, n (%) | 26 (48.2%) |
| **Race / Ethnicity**, n (%) |  |
| Latinx | 20 (39.2%) |
| White | 22 (43.1%) |
| Asian | 5 (9.8%) |
| Native Hawaiian or other Pacific Islander | 1 (2.0%) |
| Other | 3 (5.9%) |
| **BMI (kg/m^2^)**, median (IQR) | 28.6 (24.8-32.7) |
| **Comorbid conditions** |  |
| Hypertension | 5 (15.0) |
| Diabetes | 3 (6.7) |
| Chronic lung disease | 3 (3.3) |
| **Asymptomatic at baseline**, n (%) | 1 (1.9%) |
| **Duration of symptoms in days prior to randomization**, median (IQR) ^1^ | 5 (4-7) |
| **Baseline oropharyngeal RT-PCR positivity, n (%)** | 50 (87·7) |
| **Clinical Outcomes after Randomization** |  |
| Hospitalizations by day 28, n (%) | 4 (7.4%) |
| Hospitalizations and/or emergency room visits by day 28, n (%) | 7 (13.0%) |

Supplementary Figure 1

Supplemental Figure 1: Comparing transcriptomics (A), proteomics (B), T cell responses at day 28 after enrollment (C), and antibody responses (at day 28 (D) and month 7 (E) after enrollment) between the two treatment arms. (F) volcano plot showing the effect (measured as regression coefficient) and p value of Peginterferon Lambda treatment on blood transcription modules and plasma proteins.

Supplementary Figure 2

Supplementary Figure 2. (A) Characterizing the trajectory of plasma proteins and Gene Ontology pathways in COVID-19 patients. (B)The association between immune measures (plasma proteins and Gene Ontology pathways) and disease progression. We fitted regression models to test the relationship between the immune measurements and disease progression while controlling for the time after symptom onset. The t values for the disease progression term are reported. All measurements shown are significantly associated with disease progression (FDR<0.05) (C) Association between COVID-19 patient outcomes and the Gene ontology pathways that are related to interferon signaling and cell death. Stars indicate FDR<0.05.

Supplementary Figure 3

Supplementary Figure 3. We tested the goodness of fit of the regression model for characterizing the trajectory of immune measures over time. BIC (Bayesian information criterion) was used to measure the goodness of fit. Regression model with different orders of polynomial terms were tested (immune measure ~ time +time^2 +…+time^N).

Supplementary Figure 4


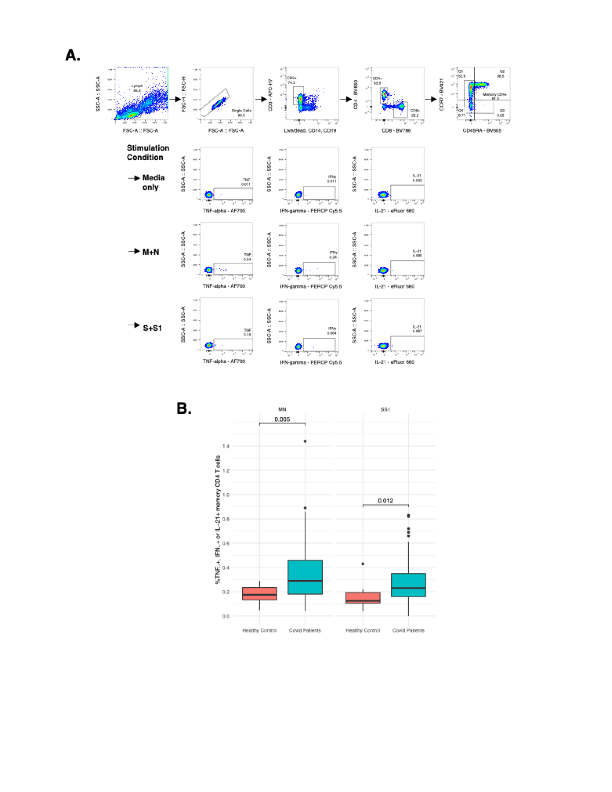


Supplementary Figure 4. Measurement of SARS-CoV-2 specific T cell responses by intracellular cytokine staining. A. Gating strategy to identify SARS-CoV-2 specific memory CD4+ T cells responsive to Membrane (M), Nucleocapsid (N), or Spike (S, S1) immunodominant peptides by detection of intracellular cytokines TNF-alpha, IFN-gamma, or IL-21. B. Comparison of SARS-CoV-2 specific T cell responses producing either TNF-alpha, IFN-gamma, or IL-21 following MN or SS1 stimulation compared to uninfected, healthy age-matched controls

Supplementary Figure 5


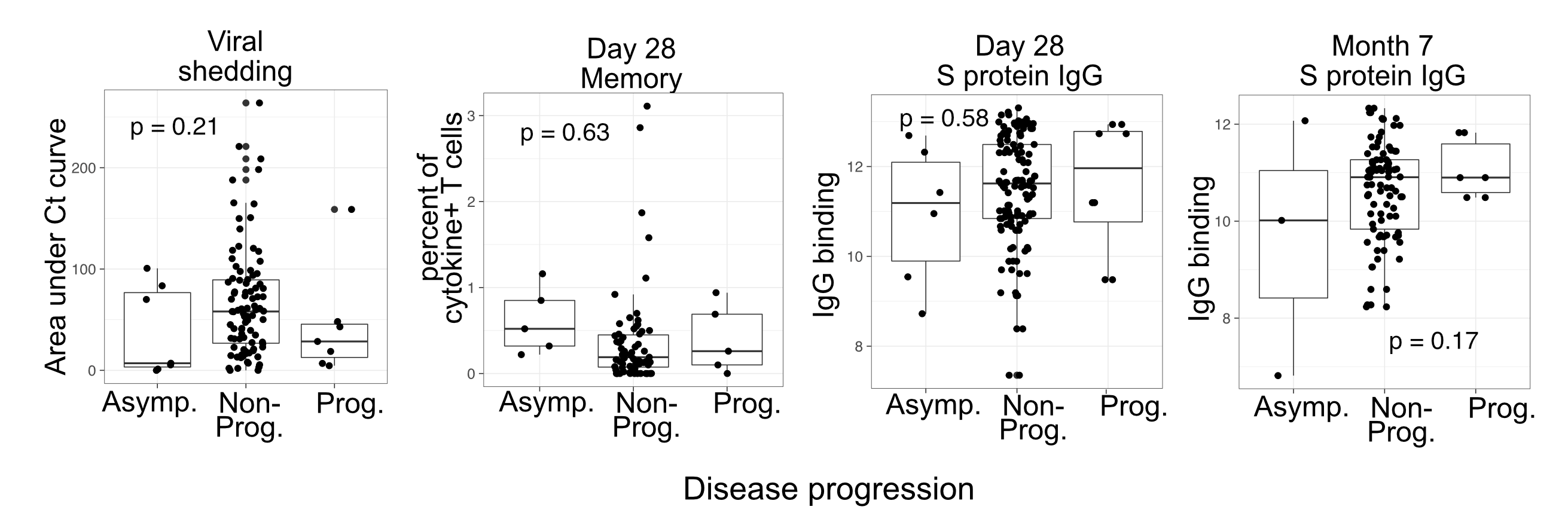


Supplementary Figure 5: Boxplots visualizing the relationship between disease progression and other COVID-19 outcomes (viral shedding, memory T cell activity, and anti-S binding IgG levels).

Supplementary Figure 6

Supplementary Figure 6. We estimated the association between immune measures (BTM and Olink proteins measures) and COVID-19 outcomes using data from both arms and from only control arm of the lambda trial. The scatter plots compare the two sets of the results.

Supplementary Figure 7

Supplementary Figure 7. We estimated the association between immune measures (BTM and Olink proteins measures) and COVID-19 outcomes using data from both lambda trial and an independent dataset from Favi trial. The scatter plots compare the two sets of the results.
